# A New Propolis Type from Changbai Mountains in North-east China: Chemical Composition, Botanical Origin and Biological Activity

**DOI:** 10.3390/molecules24071369

**Published:** 2019-04-08

**Authors:** Xiasen Jiang, Jing Tian, Yufei Zheng, Yanzheng Zhang, Yuqi Wu, Cuiping Zhang, Huoqing Zheng, Fuliang Hu

**Affiliations:** College of Animal Science, Zhejiang University, Hangzhou 310058, China; Jxsen@zju.edu.cn (X.J.); tj_1224@126.com (J.T.); iriszheng92@gmail.com (Y.Z.); 21417023@zju.edu.cn (Y.Z.); hzhzwuyuqi@163.com (Y.W.); lgzcplyx@zju.edu.cn (C.Z.); hqzheng@zju.edu.cn (H.Z.)

**Keywords:** propolis, Changbai Mountains, poplar, p-coumaric acid, benzyl *p*-coumarate

## Abstract

Propolis is a bee product with a wide range of biological activities and its chemical compounds depend highly on the type of plant accessible to the bees. The Changbai Mountains are a major mountain range in Northeast China and are one of the major bee product-producing areas in China. In this study, we evaluated the total phenolic acids and flavonoid contents as well as the antioxidant activity of propolis sampled from the Changbai Mountains area (CBM). We identified the major compounds and qualified their contents by HPLC-ESI/MS and HPLC-UV, and found that the content of *p*-coumaric acid and an unknown peak (CBE) in CBM propolis was higher than in propolis from other parts of China. The unknown compound CBE was isolated, purified, and identified as benzyl *p*-coumarate by MS and NMR. Possible plant sources of CBM propolis are *Populus davidiana* dode and *Populus simonii* Carr, which widely distributed in the Changbai Mountains area. CBM propolis is a new propolis type, that could be an excellent raw material for health foods and pharmaceuticals.

## 1. Introduction

Propolis is a popular bee product, which consists of plant resins, waxes, pollens and small amounts of enzymes [1,2]. Honeybees collect these resinous substances from various tree buds, leaves or exudates with their mandibles and mix them with wax to block holes and cracks in hives, alter the size of the hive entrance or to encapsulate dead animals [3,4]. Propolis has been used as medicine by the ancient Greeks, Romans, and Egyptians [5], and has been reported to have various biological activities such as anti-inflammatory [6], anti-oxidant [7], anti-hyperglycemic [8], antibacterial [9], and anticancer effects [10,11]. These biological activities of propolis are closely related to its variable chemical composition, and the plant sources are deemed to make a major contribution to the propolis composition. Susbtances from a lot of plants from genera such as *Populus*, *Clusia*, *Baccharis*, *Betula*, *Ulmus*, *Pinus*, *Quercus*, *Macaranga*, *Mangifera*, *Lepidosperma*, *Salix* and *Acacia* have been confirmed to be collected by honeybees and used as raw materials for propolis [4,12]. *Populus* spp. (mainly *P. tremula* L., *P. nigra* L. and its hybrid variety) have been reported in many countries around the world, such as some countries in Europe [13], United States [14,15], Canada [16], Mexico [17], and China [18,19,20].

China has a wide variety of botanical resources, many of which are potential sources of propolis. Northeast China (Heilongjiang, Jilin and Liaoning provinces), located in the mid-temperate zone, is one of the most important sources of nectar and pollen in China. The main plant sources of nectar and pollen are *Tilia amurensis* and *Tilia mandschurica*, which are common in the forests of northeast China [21]. The Changbai Mountains (CBM) are a major mountain range in Northeast China, that determines the natural boundary between China and North Korea. The flora of the CBM are diverse, including more than 127 plant genera, 1477 species of higher plants and 510 species of lower plants [22]. To our knowledge, studies on chemical composition, botanical origin and biological activity of CBM propolis have not been reported yet. This situation has slowed down the development and possible commercial utilization of CBM propolis.

In this study, we characterized the chemical constituents of different CBM propolis by HPLC and HPLC-MS, and compared them with 154 propolis from different regions of China. We also tested the total phenolics and total flavonoids of CBM propolis. One characteristic component of CBM propolis was separated and identified. We also determined its potential botanical origins. Furthermore, examinations of the anti-oxidant activity of the CBM propolis were also performed. 

## 2. Results and Discussion

### 2.1. Contents of Total Flavonoids, Total Phenolics and Antioxidant Activity of CBM Propolis

The major constituents of propolis from temperate zones are phenolic compounds such as flavonoids, phenolic acids and their esters [23]. The Folin-Ciocalteu colorimetric method was used to determine the total content of polyphenols of 21 CBM propolis samples, and total flavonoids were measured by the aluminum ion colourimetric method. The amounts of total phenolics in CBM propolis range from 215.6 ± 0.4 to 316.8 ± 1.2 mg/g and total flavonoids from 90.5 ± 2.9 to 123.1 ± 2.8 mg/g (Table 1). This result is consistent with a previous report on the total phenolics and total flavonoids content of propolis from different regions of China, which showed ranges of approximately 200–300 mg/g and 80–190 mg/g, respectively [24]. 

It has been reported that oxidative damage is related to many diseases, such as heart disease, atherosclerosis, cancer and so on [25]. Considering the high content of phenolics in CBM propolis, we have validated the anti-oxidative activities of CBM propolis by DPPH assays, and antioxidant activity is indicated by IC_50_ values. All CBM propolis samples showed strong free radical scavenging activity, with IC_50_ ranging from 170.4 ± 2.5 to 278.5 ± 2.9 μg/mL (Table 1). The relationship between antioxidant activity (IC_50_) and total polyphenol or total flavonoids contents was calculated using the SPSS software package. IC_50_ is negatively correlated with total polyphenol contents (R2 = −0.564, *P* < 0.01), but not correlated with total flavonoids (R2 = −0.303, *P* > 0.05).

### 2.2. Profiling of Samples with HPLC-UV and HPLC-ESI/MS

**Figure 1:**
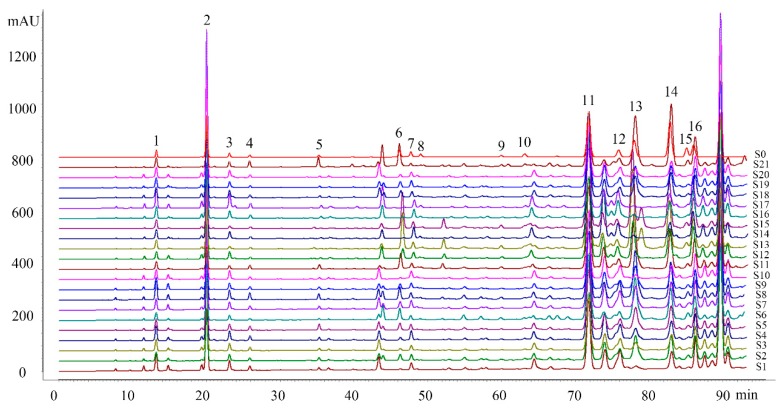
HPLC chromatograms of the CMB propolis (S1-S21) and standard solution (S0): 1. Caffeic acid; 2. *p*-Coumaric acid; 3. Ferulic acid; 4. Isoferulic acid; 5. 3,4-Dimethoxycinnamic acid; 6. Pinobanksin; 7. Naringenin; 8. Quercetin; 9. Kaempferol; 10. Apigenin; 11. Pinocembrin; 12. Benzyl caffeate; 13. 3-Oacetyl pinobanksin; 14. Chrysin; 15. CAPE; 16. Galangin.

All 21 CBM propolis samples from various sources showed similar chemical profiles (Figure 1). The 16 characteristic common peaks were identified by comparing their HPLC chemical profiles and MS information (Table 2) to those of reference compounds, after which the content of these identified compounds was tested by HPLC (Table S1). All the 16 common peaks were identified to be phenolic compounds, namely hydroxycinnamic acids (peaks 1–5), hydroxycinnamic acid esters (peaks 12 and 15), flavanones (peaks 7 and 11), flavones (peaks 10 and 14), flavonol (peaks 6, 8, 9 and 16) and a flavonol ester (peak 13), which are also common in Chinese polar-style propolis [20].

We used the software named Similarity Evaluation System for Chromatographic Fingerprint of Traditional Chinese Medicine developed by the Chinese Pharmacopoeia Committee (Version 2012.130723) to test the CBM propolis samples and the reference chromatogram. All the correlation coefficients (CC) of CBM propolis and the reference chromatogram were bigger than 0.721 (Table 1), which means the fingerprints of all CBM propolis samples are similar, and suggesting that CBM propolis have a stable quality.

### 2.3. The Difference between CBM Propolis and other Propolis from China

**Figure 2:**
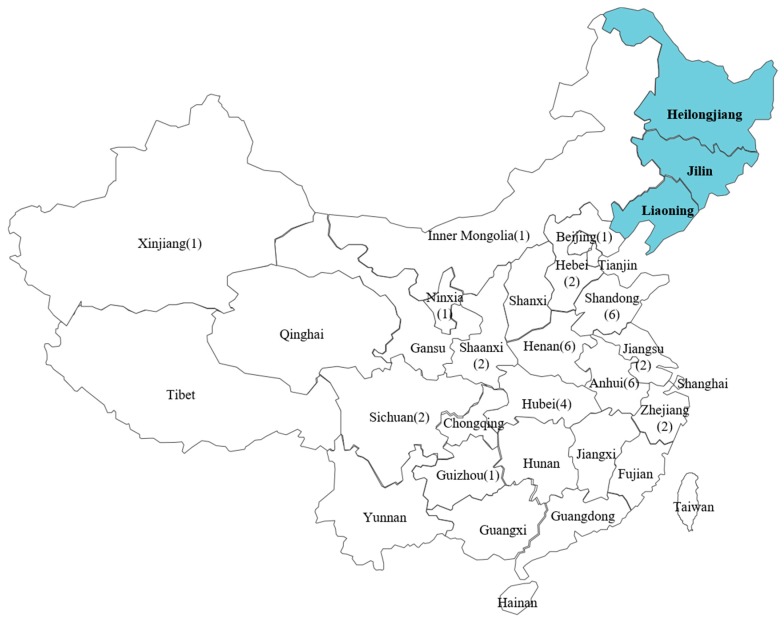
Distribution of sampling locations. Northeast China (Heilongjiang, Jilin and Liaoning provinces) was highlighted, the numbers in parentheses indicate the sample size of each province.

We also collected 49 propolis samples from different regions of 16 provinces around China (Figure 2, and analysed them using the same HPLC procedures. We evaluated 49 propolis samples and the reference chromatogram of CBM propolis were evaluated by similarity analysis, the correlation coefficients (CC) are decentralized and lower, in other words, CBM propolis samples are special and have significant differences with common Chinese propolis among the 49 samples. The CC of 32 samples are lower than 0.6, the CC of 10 samples are between 0.6–0.7, and only seven samples demonstrated a CC higher than 0.7 (Table 3). Interestingly, six of the samples with CC higher than 0.7 were collected from northeast China (Heilongjiang, Jilin and Liaoning provinces) and only one sample was collected from Zhejiang Province. We believed this can be explained by the fact that some Chinese beekeepers, especially beekeepers from the south of China like Zhejiang Province, follow the blooming season to produce honey by moving their hives to different areas of China [26], so probably this propolis was collected from bee colonies which migrated from CBM areas back to Zhejiang Province.

**Figure 3:**
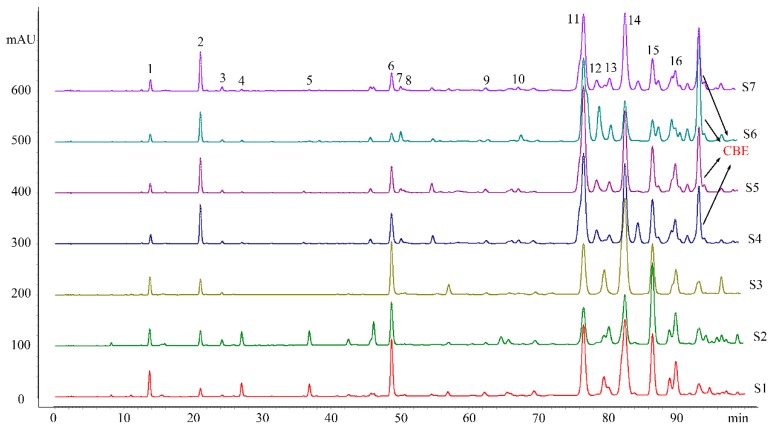
The HPLC chromatograms of common Chinese propolis (S1–S3) and the CMB propolis (S4–S7).

By contrasting the HPLC chromatograms of the CMB propolis and common Chinese propolis (Figure 3), we found the main differences depended on two peaks. The average of peak 2, *p*-coumaric acid, in CMB propolis is 28.53 mg/g but only 4.53 mg/g in common Chinese propolis. In addition, an unknown peak (CBE) appeared at the retention time of 92 min in CBM propolis. It should be noted, *p*-coumaric acid and the CBE exist in almost all 49 Chinses propolis, but the content in CBM propolis is much higher and the peak area in the HPLC spectrum is much larger. Therefore, we propose CBM propolis can be easily distinguished from ordinary Chinese propolis by the abundant presence of *p*-coumaric acid and the unknown peak (CBE).

### 2.4. Isolation and Identification of CBE in CBM Propolis

The unknown compound CBE was isolated as a light white amorphous powder, and gave a molecular formula of C_16_H_14_O_3_ based on the negative-ion ESI/MS, with a [M − H]^−^ peak occurring at *m/z* 253.10. The ^1^H-NMR spectra displayed a set of signals at *δ*_H_ 7.43 (2H, d, *J* = 8.7 Hz), 6.84 (2H, d, *J* = 8.7 Hz) ascribed to a *p*-substituted phenyl group, *δ*_H_ 7.68 (1H, d, *J* = 16.0 Hz) and 6.35 (1H, d, *J* = 16.0 Hz) due to a set of *trans*-olefinic protons, *δ*_H_ 7.35–7.40 (5H, m) suggested the presence of a single-substituted phenyl, one oxygenated methylene group (*δ*_H_ 5.25, 2H, s) and a hydroxyl proton signal (*δ*_H_ 5.44, 1H, br. s). The above NMR spectroscopic features were in agreement with the data of benzyl *p*-coumarate [27]. Accordingly, the unknown compound CBE was unambiguously identified as benzyl *p*-coumarate (Figure S2). This compound has also been detected in other propolis through different detection methods [28,29]. 

### 2.5. Authentication of CBM Propolis

**Figure 4:**
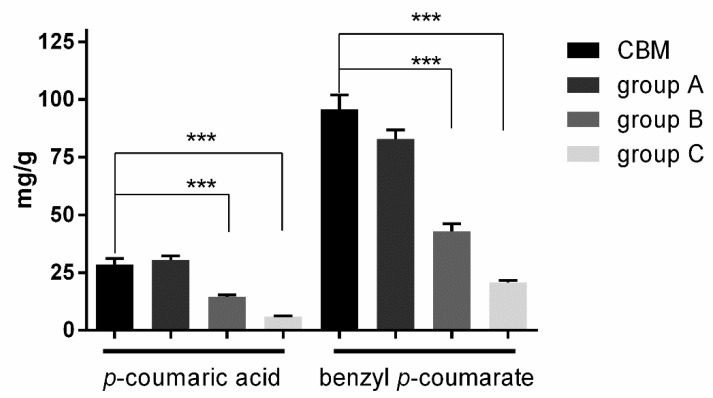
The contents of *p*-coumaric acid and benzyl *p*-coumarate in CBM and different groups propolis. Each value represents the mean ± SEM, ***P < 0.0001 by Student’s *t*-test.

Quantification of *p*-coumaric acid and benzyl *p*-coumarate as two characteristic markers was undertaken on 104 propolis samples collected from Northeast China. The samples were divided into three groups: group A, 21 samples collected from hives that only produced linden honey; group B, 20 samples collected from hives that produced linden honey and other kinds of honey; group C, 63 samples collected from hives that did not produce linden honey. Linden (*Tilia mandshurica*; *Tilla amurensis*) honey is mainly produced in the Changbai Mountain area, which meant propolis in group A were CBM propolis; propolis in group B were mixed propolis; propolis in group C were common propolis. These three groups present different averages of *p*-coumaric acid (group A: 30.53 mg/g; group B: 14.45 mg/g; group C: 2.07 mg/g) and benzyl *p*-coumarate (group A: 82.95 mg/g; group B: 42.87 mg/g; group C: 20.63 mg/g), where the average of *p*-coumaric acid is 28.53 mg/g and benzyl *p*-coumarate is 95.83 mg/g in 21 CBM propolis (Figure 4). As expected, the contents of *p*-coumaric acid and benzyl *p*-coumarate of propolis in group A are similar to CBM propolis, meanwhile, group B and group C are significantly different (***P < 0.0001). Therefore, CBM propolis can be easily distinguished from ordinary Chinese propolis by the levels of *p*-coumaric acid and benzyl *p*-coumarate.

### 2.6. The Botanical Source of the CBM Propolis

As mentioned above, bees collect resins from different plants to produce various types of propolis, that is to say, the chemical composition of propolis is mainly dependent on the plant species in the area [30]. The HPLC chemical profiles and MS information of CBM propolis demonstrated that it is a poplar-type propolis, but there are obvious differences in constituents between CBM propolis and common Chinese propolis, indicating they were collected from different poplar trees. There are many *Populus* species that have been identified as propolis sources, such as *P. nigra* L., *P. tremula* L., *P. tremuloides* Michx., *P. canadensis* Moench, *P. balsamifera* L., *P. deltoides* W. Bartram ex Marshall, and *P. trichocarpa Torr*. et Gray [4]. More than 12 plants in different genera have been found that can be sources of raw material for propolis [4,12]. Therefore, we assumed the botanical source of the CBM propolis is different *Populus* species or common *Populus* species mixed with other plants. In order to define the botanical source of the CBM propolis, the HPLC chemical profiling of the CBM propolis were compared with the chemical profiling of bud and tender leaves extracts of various plants collected from the Changbai Mountains, including *Populus* spp. (*P. davidiana* Dode, *P. davidiana* Rehd, *P. simonii* Carr), *Betula platyphylla* Suk., *Salix matsudana* Koidz., *Phellodendron amurense* Rupr. and *Pinus densiflora* Sieb.et Zucc.

**Figure 5:**
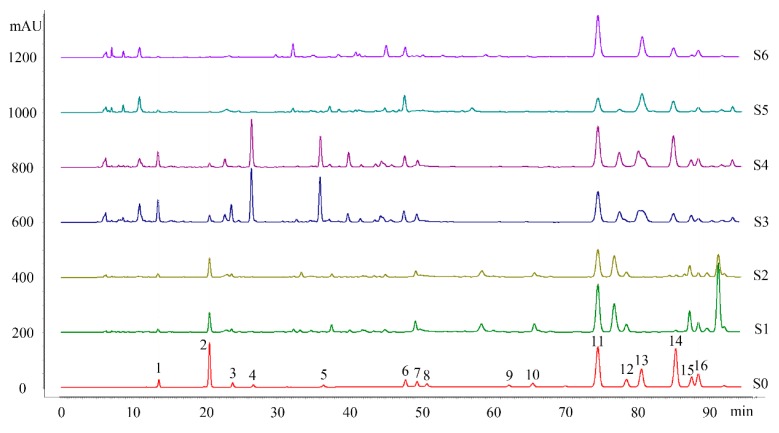
The HPLC chromatograms of the bud and tender leaves extracts of *P. davidiana* dode (S1 and S2), *P. davidiana* Rehd (S3 and S4), *P. simonii* Carr (S5 and S6) and standard solution (S0).

HPLC analysis showed a great diversity in the chemical profiles of these plant resins. *Populus* spp. extracts are rich in phenolics and their HPLC chemical profiles are similar to that of CBM propolis (Figure 5), while the profiles of other plant extracts did not show much similarity to the CBM propolis (Figure S1). The CC of CBM propolis and *P. davidiana* dode samples are 0.831 or 0.757, while *P. davidiana* Rehd samples are 0.384 or 0.345 and *P. simonii* Carr samples are 0.442 or 0.522. Then we analyzed the fingerprint of *Populus* spp. extract, we found that *P. davidiana* Dode extracts are rich in *p*-coumaric acid and benzyl *p*-coumarate, which is consistent with CBM propolis. However, we didn’t detect pinobanksin, 3-O-acetyl- pinobanksin and chrysin in this *Populus* spp. extract. In the meanwhile, *P. simonii* Carr extracts were found to be rich in pinobanksin, 3-O-acetylpinobanksin and chrysin but lack *p*-coumaric acid and benzyl *p*-coumarate. Although pinobanksin, 3-O-acetylpinobanksin and chrysin are found in most poplar propolis, including common Chinese propolis, we believe these compounds in the CBM propolis have been sourced from *P. simonii* Carr as this is the only common poplar in this area which has these resin compounds present. Taken together, we assumed that the botanical source of the CBM propolis are *P. davidiana* Dode and *P. simonii* Carr in a variable proportion. On contrast to CBM propolis, the plant source of common Chinese propolis is *P. nigra* L. and its hybrids [18,19,20]. We believed that the different botanical source is the main cause of the difference between CBM propolis and common Chinese propolis. *P. davidiana* Dode is a variety species of *P. tremula* L. in China [31], which is a common plant source of propolis in Europe and America [4]. *P. simonii* Carr is an important native poplar species in northern China belongs to the section Tacamahaca [32], this is the first time *P. simonii* Carr has been reported as a botanical source of propolis.

As mentioned, CBM propolis are rich in polyphenols and have excellent antioxidant activity (Table 1). Compared to common Chinese propolis, the contents of *p*-coumaric acid and benzyl *p*-coumarate in CBM propolis is significantly higher (Figure 4), so we can use these two compounds to verify the authenticity of CBM propolis. The botanical source of the CBM propolis are *P. davidiana* dode and *P. simonii* Carr, they are common distributed in the mountains area of northeast China [31,32]. There are many research about possibilities of propolis usage in the medicine or veterinary medicine [33]. Further research regarding the biological activity of CBM propolis and its compounds is planned.

## 3. Materials and Methods

### 3.1. Chemicals and Reagents

HPLC grade methanol was purchased from Merck (Darmstadt, Germany), analytical grade acetic acid, absolute ethanol, trichloromethane were from Chemical Reagent Factory of Zhejiang University (Hangzhou, China). Ultra-Pure water was purified by a Yjd-upws Ultra-Pure water system (Hangzhou, China). Absolute alcohol and acetic acid were purchased from Shanghai Chemical Reagent Company of Chinese Medical Group (Shanghai, China). 1,1-Diphenyl-2-picrylhydrazyl (DPPH), caffeic acid, ferulic acid, isoferulic acid, *p*-coumaric acid, 3,4-dimethoxycinnamic acid, caffeic acid phenethylester (CAPE), apigenin, galangin, chrysin, pinocembrin, quercetin, kaempferol, naringenin, were purchased from Sigma-Aldrich (St. Louis, Mo., USA), while pinobanksin, 3-O-acetylpinobanksin, and benzyl caffeate were purchased from Ningbo Haishu Apexocean Biochemicals Co., Ltd. (Ningbo, China). 

### 3.2. Collection of Propolis and Plant Material

The twenty-one CBM propolis samples (S01–S21) used in this study were harvested from apiaries located in the Changbai Mountains by local beekeepers between May 2015 and July 2016 (Table 1). We also collected the buds and tender leaves of the likely botanical origin in this area including: *Betula platyphylla* Suk., *Salix matsudana* Koidz., *Populous davidiana* Rehd, *Populus davidiana* Dode, *Populous simonii* Carr, *Phellodendron amurense* Rupr, *Pinus densiflora* Sieb.et Zucc. In addition, 49 Chinese propolis samples collected in different provinces were tested to compare with CBM propolis and 104 other propolis samples were collected in Northeast China (Heilongjiang, Jilin and Liaoning provinces) to verify the differences between common Chinese propolis and CBM propolis using this method.

The frozen propolis samples were extracted as reported previously [20]. The raw propolis and plant material were extracted with 95% hydroalcoholic solution in an ultrasonic water bath for 45 min. The resulted mixture was filtered and the residue was re-extracted twice under the same conditions. The filtrates were combined, kept at 4 °C overnight and then filtered to remove insoluble matter. After that, the filtered solution was evaporated to dryness. The dry residue powder was dissolved with ethanol to obtain solutions at 20 mg/mL and stored at 4 °C until use. 

### 3.3. HPLC and HPLC-ESI/MS Analysis

Chromatographic analysis was performed with an Agilent 1200 Series (Agilent Technologies, Inc., Santa Clara, CA, USA) equipment, using a Sepax HP-C18 column (150 × 4.6 mm, 5 μm; Sepax Technologies, Inc., Newark, DE, USA) and maintained at 33 °C. The mobile phase consisted of both aqueous phase A, 1% acetic acid and organic phase B, methanol at a constant flow rate of 1 mL/min. The gradient elution was adjusted as follows: 15% to 35% (B) from 0 to 30 min, 35% to 44% (B) from 30 to 46 min, 44% to 50% (B) from 46 to 70 min, 50% to 52% (B) from 70 to 77 min, 52% to 60% (B) from 77 to 92 min, 60% to 75% (B) from 92 to 115 min, 75% to 100% (B) from 115 to 125 min and finally 100% to 15% (A) from 125 to 135 min. Each sample was purified with 0.45 μm filters (5 μL) then injected through an automatic sampler system and monitored at 280 nm. The above HPLC system was also carried out using an Agilent 6430 QQQ MS (Agilent Technologies, Inc.) instrument equipped with an electronic spray ionization (ESI) interface with the following operating conditions: drying gas (N2) flow rate, 9.0 mL/min; drying gas temperature, 350 °C; nebulizer, 35 psig; capillary, 4000 V; fragmentor voltage, 135 V. The mass spectra were analyzed in negative ion mode and comparison with previously published data.

### 3.4. Antioxidant Capacity

Fresh DPPH stock solution was prepared by dissolving 3 mg DPPH in 10 mL ethanol (0.3 mg/mL), sealed and stored in the refrigerator at 4 °C. The DPPH antioxidant activity was determined as our earlier research [34]. In brief, 100 μL DPPH working solution was mixed with 100 μL propolis solutions in different concentrations in a 96-well plate. The absorbance of the reaction solutions was read at 517 nm after incubating for 30 min in the dark. 

### 3.5. Determinations of Total Flavonoids and Total Phenolics

Total flavonoids content (TFC) was measured by aluminum ion chromogenic method with minor modifications (refer to quercetin) [34] and the amount of total phenolics (refer to gallic acid) was determined by the modified Folin–Ciocalteau method [35]. A detailed procedure is available in our recent publication [36].

### 3.6. Isolation and Identification of the Unknown Compound CBE

The ethanol extract (*ca*. 30 g) of CBM propolis was fractionated by silica gel CC successively eluted with a gradient of increasing chloroform in methanol (10:1→0:1, *v/v*) and produced fractions A–E, the fraction B with a higher purity (>70%) of the target compound was subjected to Sephadex LH-20 (CHCl_3_/MeOH, 1:1, *v/v*, GE Healthcare Bio-Sciences AB, Uppsala, Sweden) and then separated further by preparative HPLC (Agilent 1260, Daisogel C-18 column (250 × 20 mm, 10 μm), MeOH/H_2_O = 27:73, *v/v*; 12 mL/min, 25 °C) to afford high purity of target product. The HPLC-ESI /MS procedures were implemented as above and the ^1^H NMR spectra were carried out on a Bruker AV-600 instrument (Bruker, Karlsruhe, Germany) with deuterated solvent signals used as internal standards to establish the structures of unknow compounds by comparison with reference data.

### 3.7. Statistical Analysis

Data are expressed as mean ± SD and each value is representative of at least three independent experiments. The correlation coefficients (CC) of different samples were performed using Similarity Evaluation System for Chromatographic Fingerprint of Traditional Chinese Medicine. The CC value of all samples relative to standard chromatogram would be calculated by using the cosine value of the angle [37]. The IC_50_ was performed by SPSS statistics software (SPSS for Windows 20.0, SPSS Inc., Chicago, IL, USA). Student’s t-test was employed using GraphPad Prism 6 (GraphPad, San Diego, CA, USA) to confirm the significance when two groups were compared. (*P < 0.05; **P < 0.01).

## 4. Conclusions

As far as we know, our study is the first relevant report on the chemical composition, botanical origin and biological activity of propolis collected from Changbai Mountains. CBM propolis belongs to poplar-type propolis, but it has obvious differences in chemical composition compared to propolis from other regions of China. The botanical source of the CBM propolis were *P. davidiana* dode and *P. simonii* Carr. *p*-Coumaric acid and benzyl *p*-coumarate could be used to distinguish CBM propolis from common Chinese propolis. Furthermore, CBM propolis was rich in polyphenols and had excellent antioxidant activity, which revealed the potential usage of CBM propolis as a good raw material for health foods and pharmaceuticals.

## Figures and Tables

**Table 1:** Total phenolic, total flavonoid, antioxidant activity and correlation coefficients of CBM propolis. Note: Data are shown as the mean ± SD (*n* = 3). GAE, gallic acid equivalent; QE, quercetin equivalent.

Sample No.	Origin (City/Province)	Collection Date	Total Flavonoids (mg/g, QE)	Total Phenolics (mg/g, GAE)	DPPH Scavenging Activity (IC_50_,μg/mL)	Correlation Coefficients
1	Jixi, Heilongjiang	May 2015	117.8 ± 5.9	284.2 ± 0.4	231.1 ± 4.2	0.957
2	Jixi, Heilongjiang	May 2015	105.2 ± 5.7	253.0 ± 6.8	222.5 ± 9.2	0.982
3	Mudanjiang, Heilongjiang	May 2015	95.5 ± 6.0	243.4 ± 3.3	256.7 ± 9.0	0.968
4	Jixi, Heilongjiang	May 2015	113.6 ± 2.1	285.4 ± 3.7	233.2 ± 4.2	0.986
5	Jixi, Heilongjiang	May 2015	121.7 ± 1.4	263.2 ± 1.5	231.2 ± 10.5	0.983
6	Shuangyashan, Heilongjiang	May 2016	114.3 ± 5.4	262.7 ± 1.9	267.5 ± 6.3	0.904
7	Qiqihaer, Heilongjiang	May 2016	111.4 ± 4.4	286.9 ± 0.4	221.2 ± 9.4	0.984
8	Qitaihe, Heilongjiang	May 2016	105.4 ± 4.5	316.8 ± 1.2	204.5 ± 1.1	0.939
9	Jixi, Heilongjiang	May 2016	98.3 ± 4.8	215.6 ± 0.4	278.5 ± 2.9	0.982
10	Jixi, Heilongjiang	May 2016	123.1 ± 2.8	298.1 ± 1.6	193.6 ± 8.1	0.966
11	Shuangyashan, Heilongjiang	May 2016	106.8 ± 6.0	273.7 ± 3.7	215.5 ± 2.8	0.879
12	Shuangyashan, Heilongjiang	May 2016	108.3 ± 2.7	255.8 ± 1.6	184.3 ± 4.7	0.928
13	Qitaihe, Heilongjiang	May 2016	114.1 ± 5.2	299.6 ± 0.8	188.8 ± 2.4	0.721
14	Mudanjiang, Heilongjiang	May 2016	105.5 ± 2.8	275.6 ± 2.9	170.4 ± 2.5	0.955
15	Jixi, Heilongjiang	May 2016	106.9 ± 1.9	268.7 ± 4.2	183.9 ± 7.4	0.721
16	Haerbin, Heilongjiang	July 2016	90.5 ± 2.9	265.4 ± 3.9	188.3 ± 3.5	0.949
17	Jiilin, Jilin	July 2016	123.1 ± 7.5	300.6 ± 1.6	171.9 ± 2.0	0.902
18	Jiilin, Jilin	July 2016	97.7 ± 3.7	257.1 ± 4.0	234.4 ± 0.7	0.986
19	Dunhua, Jilin	July 2016	101.1 ± 2.1	247.5 ± 1.6	263.1 ± 3.3	0.983
20	Jiilin, Jilin	July 2016	106.7 ± 5.4	250.3 ± 1.5	248.6 ± 2.9	0.966
21	Jiilin, Jilin	July 2016	108.9 ± 1.6	245.2 ± 2.4	262.9 ± 3.0	0.778

**Table 2:** Composition data for CBM propolis.

Peak	Compounds	MW	[M − H]^−^	Retention Time (min)
1	caffeic acid	180	179.1	13.694
2	*p*-coumaric acid	164	163.1	20.693
3	ferulic acid	194	193.1	23.646
4	isoferulic acid	194	193.1	26.474
5	3,4-dimethoxycinnamic acid	208	207.1	35.903
6	pinobanksin	272	271.1	46.894
7	naringenin	272	271.1	48.358
8	quercetin	302	301.1	52.910
9	kaempferol	286	285.1	60.956
10	apigenin	270	269.1	64.924
11	pinocembrin	256	255.1	73.517
12	benzyl caffeate	270	269.1	76.338
13	3-O-acetylpinobanksin	314	313.1	79.337
14	chrysin	254	253.1	84.079
15	CAPE	284	283.1	86.048
16	galangin	270	269.1	87.329

**Table 3:** The Correlation coefficients between 49 Chinese and reference chromatogram of CBM propolis.

Sample No.	Correlation Coefficients	Samples No.	Correlation Coefficients	Samples No.	Correlation Coefficients	Samples No.	Correlation Coefficients	Samples No.	Correlation Coefficients
Anhui1	0.566	Shandong2	0.566	Liaoning1	0.586	Henan2	0.596	Hubei4	0.618
Anhui2	0.427	Shandong3	0.551	Liaoning2	0.596	Henan3	0.663	Zhejiang1	0.848
Anhui3	0.415	Shandong4	0.54	Liaoning3	0.574	Henan4	0.591	Zhejiang2	0.612
Anhui4	0.522	Shandong5	0.505	Heilongjiang1	0.533	Henan5	0.599	Ningxia	0.503
Anhui5	0.509	Shandong6	0.505	Heilongjiang2	0.758	Henan6	0.631	Xinjiang	0.664
Anhui6	0.563	Shaanxi1	0.51	Heilongjiang3	0.798	Jiangsu1	0.596	Beijing	0.668
Guizhou	0.598	Shaanxi2	0.491	Heilongjiang4	0.907	Jiangsu2	0.538	Inner Mongolia	0.577
Sichuan1	0.651	Jilin1	0.672	Heilongjiang5	0.496	Hubei1	0.684	Hebei1	0.559
Sichuan2	0.643	Jilin2	0.745	Heilongjiang6	0.819	Hubei2	0.696	Hebei2	0.469
Shandong1	0.531	Jilin3	0.741	Henan1	0.545	Hubei3	0.486		

## Supplementary Materials

The following are available online. Figure S1: The HPLC chromatograms of the bud and tender leaves extracts of *Betula platyphylla* Suk.(S1), *Salix matsudana* Koidz.(S2), *Phellodendron amurense* Rupr.(S3), *Pinus densiflora* Sieb.et Zucc.(S4) and standard solution (S0); Figure S2. Structures of benzyl *p*-coumarate; Table S1. The content of common compounds in different CBM propolis.
