# Supplementary material for: A New Propolis Type from Changbai Mountains in North-east China: Chemical Composition, Botanical Origin and Biological Activity
Source: Molecules. 2019 Apr 8;24(7):1369. doi: 10.3390/molecules24071369 (PMC6479873; doi:10.3390/molecules24071369)
Supplement: Supplementary file 1 [file molecules-24-01369-s001.pdf]

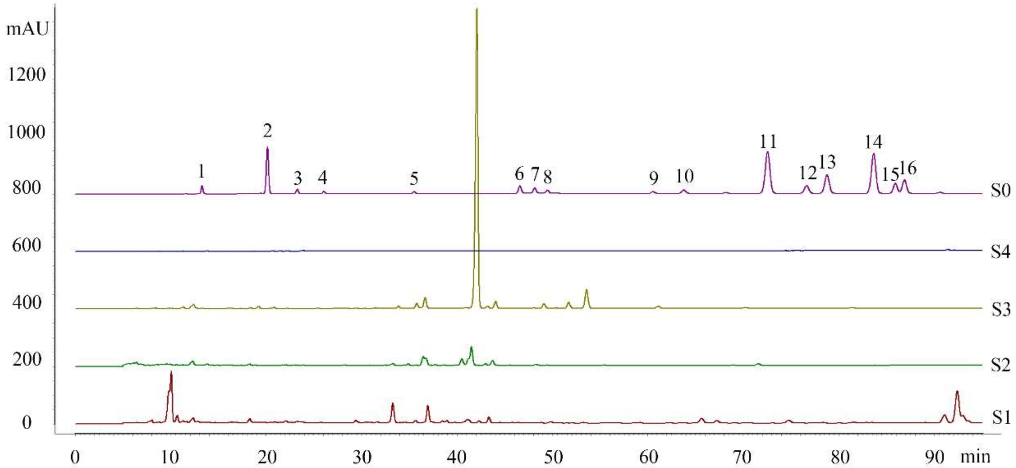

Figure S1. The HPLC chromatograms of the bud and tender leaves extracts of *Betula platyphylla* Suk.(S1), *Salix matsudana* Koidz.(S2), *Phellodendron amurense* Rupr.(S3), *Pinus densiflora* Sieb.et Zucc.(S4) and standard solution (S0).

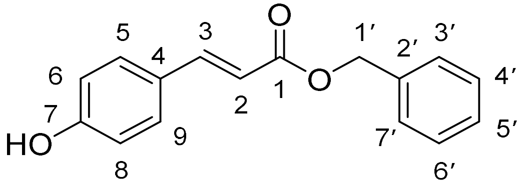

Figure S2. Structures of benzyl *p*-coumarate

Table S1. The content of common compounds in different CBM propolis.

Samples No.	Amount (mg/g, mean ± SD)															
	Caffeic acid	*p*-Coumaric acid	Ferulic acid	Isoferulic acid	3,4-Dimethoxycinnamic acid	Pinobanksin	Naringenin	Quercetin	Kaempferol	Apigenin	Pinocembrin	Benzyl caffeate	3-Oacetyl pinobanksin	Chrysin	CAPE	Galangin
S1	6.34±0.06	45.23±0.47	5.65±0.06	2.19±0.01	1.34±0.01	0.65±0.01	0.77±0.15	0.96±0.25	0.85±0.02	0.74±0.03	54.45±0.56	34.3±0.37	2.52±0.03	9.50±0.1	1.38±0.02	29.09±0.32
S2	3.19±0.04	19.54±0.20	3.33±0.04	1.43±0.02	1.41±0.02	3.46±0.04	0.80±0.01	0.99±0.01	1.13±0.01	1.63±0.02	57.12±0.69	21.87±0.03	18.22±0.23	14.06±0.17	4.86±0.06	24.67±0.29
S3	4.19±0.01	29.95±0.13	4.33±0.01	1.72±0.01	1.14±0.00	1.14±0.00	0.88±0.03	1.13±0.05	0.94±0.00	1.17±0.01	42.93±0.11	21.53±0.08	5.64±0.01	8.88±0.03	1.91±0.00	23.46±0.08
S4	5.54±0.08	31.41±0.51	5.5±0.08	2.34±0.01	1.45±0.02	1.62±0.03	1.04±0.02	1.38±0.03	1.31±0.01	1.55±0.01	67.00±0.96	29.24±0.45	8.69±0.13	11.11±0.16	2.56±0.04	26.86±0.04
S5	4.20±0.06	23.38±0.29	3.5±0.05	2.04±0.04	3.00±0.04	3.87±0.06	0.77±0.01	0.96±0.01	1.3±0.02	1.71±0.03	52.16±0.73	17.53±0.04	16.98±0.23	13.83±0.19	4.38±0.05	23.19±0.03
S6	2.80±0.01	18.82±0.09	2.67±0.00	0.87±0.00	0.71±0.01	6.7±0.03	1.13±0.10	1.52±0.16	2.31±0.01	1.22±0.39	58.55±0.03	9.60±0.20	30.32±0.05	18.18±0.08	6.98±0.05	26.46±0.07
S7	4.05±0.00	25.8±0.06	4.13±0.01	1.78±0.01	1.52±0.00	4.00±0.37	4.51±0.65	1.31±0.25	1.31±0.01	1.34±0.03	63.83±0.13	31.06±0.03	15.12±0.06	15.15±0.02	6.30±0.00	29.59±0.02
S8	8.03±0.03	30.09±0.08	5.24±0.02	3.5±0.01	2.82±0.02	6.62±0.02	2.94±0.02	1.70±0.02	2.40±0.01	1.14±0.00	71.14±0.22	32.45±0.12	36.28±0.11	21.36±0.09	7.34±0.03	32.22±0.12
S9	4.45±0.01	17.2±0.04	3.27±0.43	1.43±0.00	1.26±0.00	2.96±0.13	3.26±0.30	1.63±0.09	1.12±0.01	1.28±0.01	49.44±0.11	10.85±0.07	16.65±0.03	12.46±0.02	3.91±0.01	20.02±0.03
S10	3.59±0.02	31.38±0.13	5.09±0.02	1.28±0.01	0.71±0.00	0.80±0.01	4.78±0.02	0	0.91±0.01	1.02±0.00	54.30±0.22	23.23±0.11	2.22±0.01	7.60±0.03	1.52±0.01	25.12±0.09

S11	2.79±0.01	8.61±0.01	1.59±0.01	1.98±0.00	2.26±0.00	8.25±0.02	2.39±0.01	2.14±0.01	2.11±0.01	1.2±0.03	73.29±0.09	12.19±0.45	36.57±0.29	21.97±0.04	5.13±0.01	32.81±0.04
S12	2.66±0.01	32.06±0.07	3.96±0.01	0.9±0.00	0.60±0.00	5.40±0.03	2.47±0.08	2.04±0.06	2.00±0.00	0	72.35±0.09	25.36±0.04	6.19±0.03	17.23±0.02	0	40.33±0.05
S13	3.19±0.37	20.85±0.05	2.21±0.04	0.88±0.01	0.60±0.01	19.89±0.17	2.61±0.20	1.43±0.09	2.66±0.47	1.27±0.83	87.33±0.11	19.53±0.13	5.00±0.85	24.55±5.17	5.93±0.03	39.55±0.58
S14	3.44±0.01	28.3±0.10	3.87±0.02	1.01±0.01	0.58±0.00	0.51±0.00	5.44±0.02	2.89±0.01	1.23±0.00	1.23±0.00	89.06±0.36	65.15±0.25	8.86±0.03	8.67±0.04	1.76±0.02	28.17±0.12
S15	4.17±0.04	36.55±0.46	3.31±0.04	1.68±0.08	1.51±0.02	1.20±0.01	3.04±0.44	1.83±0.67	1.79±0.84	1.78±0.06	67.20±0..81	25.17±0.11	7.23±2.02	16.39±8.46	2.4±0.03	31.85±5.06
S16	4.41±0.01	33.17±0.07	4.07±0.01	1.71±0.00	0.86±0.00	0.69±0.00	3.85±0.03	2.42±0.12	1.11±0.03	1.32±0.01	61.07±0.14	37.62±0.08	10.30±0.01	8.47±0.02	2.06±0.02	29.75±0.05
S17	7.4±0.08	65.75±0.75	8.03±0.09	2.4±0.02	1.33±0.01	0.30±0.00	3.38±0.05	3.28±0.03	1.12±0.03	1.96±0.01	45.32±0.50	22.9±0.25	10.15±0.11	7.86±0.09	2.17±0.03	33.23±0.34
S18	4.05±0.01	30.83±0.09	4.12±0.02	1.41±0.01	0.97±0.00	3.83±0.12	2.40±0.20	2.05±0.12	1.07±0.00	1.52±0.01	53.91±0.32	18.63±0.07	14.38±0.05	14.4±0.05	3.40±0.01	23.42±0.09
S19	3.76±0.01	18.97±0.07	3.67±0.01	1.02±0.01	0.78±0.02	3.34±0.01	2.87±0.01	1.74±0.01	0.95±0.00	1.19±0.11	60.24±0.14	17.8±0.02	13.92±0.02	12.54±0.03	3.07±0.01	20.44±0.06
S20	3.92±0.01	37.3±0.05	4.1±0.01	1.43±0.00	0.77±0.01	1.62±0.06	2.13±0.12	2.72±0.07	0.86±0.00	1.15±0.18	43.54±0.11	17.41±0.93	5.32±0.18	9.42±0.03	1.51±0.02	21.8±0.07
S21	3.52±0.06	13.94±0.23	2.99±0.05	3.12±0.04	5.12±0.08	13.43±0.19	0.48±0.12	0.94±0.11	2.18±0.03	2.67±0.06	48.65±0.72	14.42±0.24	37.53±0.55	34.99±0.62	6.84±0.28	37.82±0.67
